# Malnutrition Mortality Among Older Adults by County and Race and/or Ethnicity in the United States, 2000–2019

**DOI:** 10.1111/jgs.70042

**Published:** 2025-09-12

**Authors:** Juliana Teruel Camargo, Jessica Otero Machuca, Amanda S. Hinerman, Erik J. Rodriquez, Christian S. Alvarez, George A. Mensah, Stephanie M. George, Frank Bandiera, Zhuochen Li, Dillon O. Sylte, Yekaterina O. Kelly, Theresa A. McHugh, Mathew M. Baumann, Michael Celone, Demewoz Haile, Wichada La Motte‐Kerr, Christopher J. L. Murray, Laura Dwyer‐Lindgren, Ali H. Mokdad, Eliseo J. Pérez‐Stable

**Affiliations:** ^1^ Epidemiology and Community Health Branch, Division of Intramural Research National Heart, Lung, and Blood Institute, National Institutes of Health Bethesda Maryland USA; ^2^ Division of Intramural Research, National Institute on Minority Health and Health Disparities National Institutes of Health Bethesda Maryland USA; ^3^ Center for Translation Research and Implementation Science, National Heart, Lung, and Blood Institute National Institutes of Health Bethesda Maryland USA; ^4^ Office of Dietary Supplements National Institutes of Health Bethesda Maryland USA; ^5^ Division of Behavioral and Social Research National Institute on Aging, National Institutes of Health Bethesda Maryland USA; ^6^ Institute for Health Metrics and Evaluation University of Washington Seattle Washington USA; ^7^ Department of Health Metrics Sciences University of Washington Seattle Washington USA

**Keywords:** malnutrition, medical geography, mortality, older adults, population groups

## Abstract

**Background:**

Older adults are at an increased risk of malnutrition due to chronic diseases and social vulnerabilities. This study estimates protein‐energy malnutrition mortality rates among adults aged 65–74 and ≥ 75 by race and ethnic population group and county.

**Methods:**

We analyzed death data from the National Vital Statistics System and population data from the National Center for Health Statistics from 2000 to 2019. We calculated county‐level mortality rates using small‐area estimation methods, adjusting for misclassifications in death certificates. The primary outcome was deaths attributed to malnutrition. The exposures were to populations (American Indian/Alaskan Native [AIAN], Asian, Black, Hispanic/Latino, and White) and the county.

**Results:**

From 2000 to 2019, malnutrition mortality rates increased in individuals aged ≥ 75 from 19.5 (95% uncertainty interval [UI]: 18.8–20.1) to 49.2 (48.4–50.0) deaths per 100,000, and in those aged 65–74 from 2.2 (2.0–2.3) to 4.6 (4.4–4.7). In 2019, Black individuals had the highest national mortality rates: 60.8 (58.2–63.3) for ≥ 75 years and 7.7 (7.3–8.2) for 65–74 years. In 2019, a county in Georgia had the highest rate for White individuals aged ≥ 75 at 334.9 (236.6–464.8), and a county in Montana had the highest for AIAN individuals aged 65–74 at 34.9 (13.1–72.0). Counties in the New York metro had the lowest mortality rates across all population groups and ages.

**Conclusion:**

Malnutrition mortality rates have increased among older adults, varying by geography and population group, underscoring the need for targeted nutritional interventions.

**Summary**

- Key points
  ○ In 2019, the protein‐energy malnutrition mortality rate for adults aged 75 and older was 49.2 deaths per 100,000, a 152.7% increase since 2000.
  ○ Black adults experienced the highest malnutrition mortality rates, followed by American Indian/Alaskan Native, White, Latino, and Asian adults.
  ○ Alabama, Arkansas, Arizona, Georgia, Louisiana, the Carolinas, and Utah counties had the highest malnutrition mortality rates.
- Why does this paper matter?
  ○ This study reports an increase in mortality from protein‐energy malnutrition among older adults, particularly those aged 75 and older, with a disproportionate burden among Black and American Indian/Alaskan Native populations and in the South and Southwest of the country.

## 1 Introduction

Malnutrition, defined as undernutrition due to disease processes or medical conditions, is an important clinical concern in older adults that needs to be addressed to support healthy aging [1]. Malnutrition is common in adults over the age of 65 [2], particularly among those who are hospitalized [3] or living in residential care facilities [4]. As individuals age, physiological functions decline and, combined with reduced resilience, this process increases malnutrition risk and elevates the risk of morbidity and premature mortality [5].

Malnutrition among older adults is often disease‐related [6] and preceded by acute and/or chronic illnesses that disrupt food intake [7]. These illnesses adversely impact eating through changes in appetite regulation, gastric emptying, and skeletal muscle integrity, as well as by impairing sensory and cognitive functions [7, 8, 9, 10]. Malnutrition may also result from non‐disease‐related factors such as individual and community socioeconomic determinants and environmental developments, which impact food availability and access, resulting in inadequate food intake [6, 10, 11, 12, 13]. A forensic study conducted in Japan revealed that 70% of deaths in malnourished older adults were attributed to disease, while the remaining 30% of deaths were linked to non‐disease‐related factors [14]. Despite these findings, there is a gap in research exploring demographic trends in older adults' malnutrition mortality. The physical consequences of malnutrition include increased risk of muscle weakness, compromised immune function, and susceptibility to infection. These changes can lead to increased and prolonged hospitalizations, culminating in increased mortality rates [15].

Between 1999 and 2020, few studies have explored the malnutrition mortality rates by population groups and county of residence [16, 17, 18]. To address this gap, we estimated malnutrition mortality rates by county and population groups from 2000 to 2019. This study aimed to characterize the burden of malnutrition for individuals aged 65 to 74 years and≥ 75 years by population groups, thereby informing efforts to address and alleviate this condition in the populations most affected by it.

## 2 Methods

Previously established methods for estimating specific mortality for race and/or ethnicity by county were employed [19, 20]. Mortality rates due to malnutrition were estimated from 2000 to 2019 using death record data from the National Vital Statistics System (NVSS) and population estimates from the National Center for Health Statistics (NCHS). We compiled data on deaths and populations by county, tabulated into age group (0, 1–4, 5–9, 10–14, 15–19, 20–24, 25–29, 30–34, 35–39, 40–44, 45–49, 50–54, 55–59, 60–64, 65–69, 70–74, 75–79, 80–84, 85+ years of age), race and/or ethnicity, and year. The racial and/or ethnic populations used were single‐race populations. Therefore, we used the primary (or bridged) race imputed by the NCHS for decedents identified as having multiple racial identities [21].

The classification of race and/or ethnicity was unified into a single grouping of five distinct populations: American Indian or Alaska Native (AIAN), Asian or Native Hawaiian or Pacific Islander (Asian), Black (Black), Latino or Hispanic (Latino), and White (White). Due to limitations in the available data set, the Asian and Native Hawaiian and Pacific Islander (NHPI) populations were merged for the analysis. We identify this combined population as Asian, recognizing that the estimates primarily reflect the larger Asian population (21.7 million persons) compared to NHPI (1.2 million persons) [22].

For this analysis, we used a previously developed mapping of counties to temporally stable geographical units [23]. This mapping process reduced the number of areas under analysis from 3143 to 3110 counties or combined county units, denoted as counties throughout the manuscript.

We used the cause list and hierarchy developed for the Global Burden of Diseases, Injuries and Risk Factors Study (GBD) 2021 [24] and the ICD‐10 codes linked to GBD causes. Only deaths with protein‐energy malnutrition (henceforth malnutrition) as an underlying cause were considered (ICD codes E40–E46.9 and E64.0). Previously established methods were used to reassign codes originally assigned as an underlying cause of death code that referred to an intermediate or immediate cause of death that was otherwise implausible or insufficiently specific to the likely true underlying causes of death [24, 25]. Across all causes, the percentage of deaths with these intermediate or immediate, implausible, or insufficiently specific codes was similar by population groups, ranging from 25.5% for the Latino population to 29.4% for the Black population. For malnutrition specifically, this redistribution process increased the number of deaths assigned to malnutrition by 22.1% overall, ranging from 12.7% for the AIAN population to 23.2% for the White population.

We utilized the United States Department of Agriculture (USDA) Rural–Urban Continuum Codes to classify counties as metropolitan or nonmetropolitan [26]. Counties were categorized according to their metro or nonmetro status, as defined by the Office of Management and Budget. Metropolitan areas include central counties containing urban centers with populations of 50,000 or more people and surrounding counties with strong economic ties to these areas. Nonmetropolitan counties are classified in three categories based on urban population size: (1) 20,000 or more, (2) 5000 to 20,000, and (3) fewer than 5000. Nonmetro counties are further divided based on whether they are adjacent to a metropolitan area and have at least 2% of their employed workforce commuting to central metropolitan counties [26].

### 2.1 Statistical Analysis

The statistical analysis employed small‐area estimation models to estimate malnutrition mortality rates across counties, race and/or ethnicity, age groups, and years. These models aimed to estimate the underlying mortality rate while minimizing random variability. We utilized the Template Model Builder package [27] in R version 3.6.1 to construct the models. Subsequently, we generated 1000 iterations of the mortality rate based on the approximated posterior distribution post model fitting. Then, we adjusted the mortality rate iterations obtained from the small‐area model using previously published race and/or ethnicity misclassification ratios [21]. These previously published misclassification ratios were calculated as the ratio of deaths among individuals of a specific racial and/or ethnic group, determined by self‐report, to deaths among individuals of the same racial/ethnic group as indicated on death certificates [21]. Finally, to ensure coherence across causes and maintain consistency in the estimated mortality rate for each county following misclassification adjustments, we conducted post hoc calibration employing a two‐stage iterative proportional fitting algorithm. The final estimates were calculated based on the mean of the 1000 iterations, while the 95% uncertainty intervals (UI) were determined from the 2.5th and 97.5th percentiles of these iterations. Age‐standardized mortality rates were computed for ages 55–64 (combining 55–59 and 60–64, Figures S1 and S2), 65–74 (combining 65–69 and 70–74), and ≥ 75 (combining 75–79, 80–84, ≥ 85) using the age distribution of the US population as outlined in the 2010 U.S. census.

We withheld the annual mortality rate estimates from display for counties and racial and/or ethnic groups where the mean annual total population was below 1000, as model performance declined below this threshold [19]. Consequently, we provide estimates for 3079 (out of 3110) counties for the total population, 474 for the AIAN population, 667 for the Asian population, 1488 for the Black population, 1478 for the Latino population, and 3051 for the White population. More than 97% of the population in each racial and/or ethnic group, except AIAN, resided in counties with unmasked estimates; 82% of the AIAN population lived in counties with unmasked estimates. To display county‐level results in 2019, we capped the color scale at approximately the 1st and 99th percentile mortality rates observed across all counties and race and/or ethnicity populations with unmasked estimates.

This study adheres to the Guidelines for Accurate and Transparent Health Estimates Reporting (Table S3) [28]. Institutional review board approval was obtained from the University of Washington for this research.

## 3 Results

**FIGURE 1:**
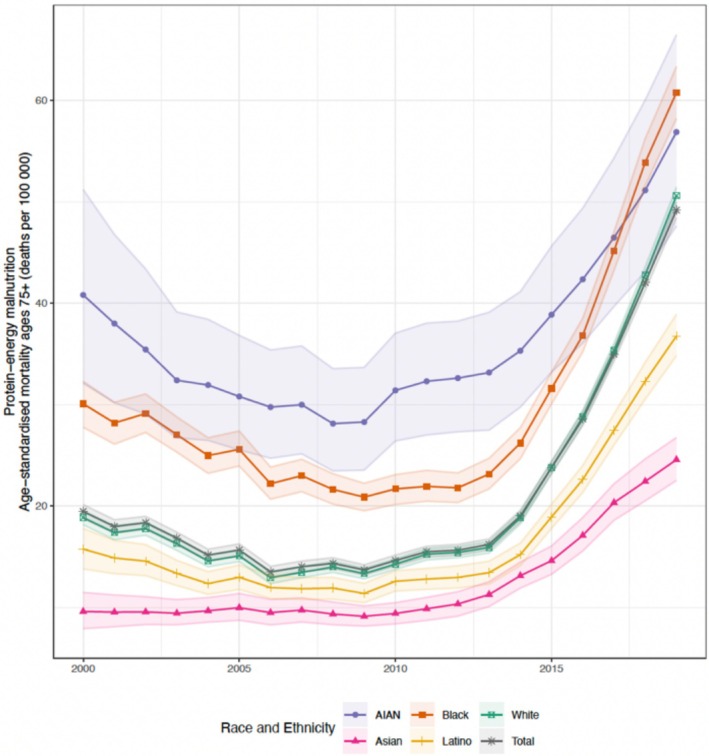
National estimated age‐standardized protein‐energy malnutrition mortality rates for individuals aged ≥ 75, by year and race and/or ethnic population groups, 2000–2019, U.S. Shaded areas are 95% uncertainty intervals.

**FIGURE 2:**
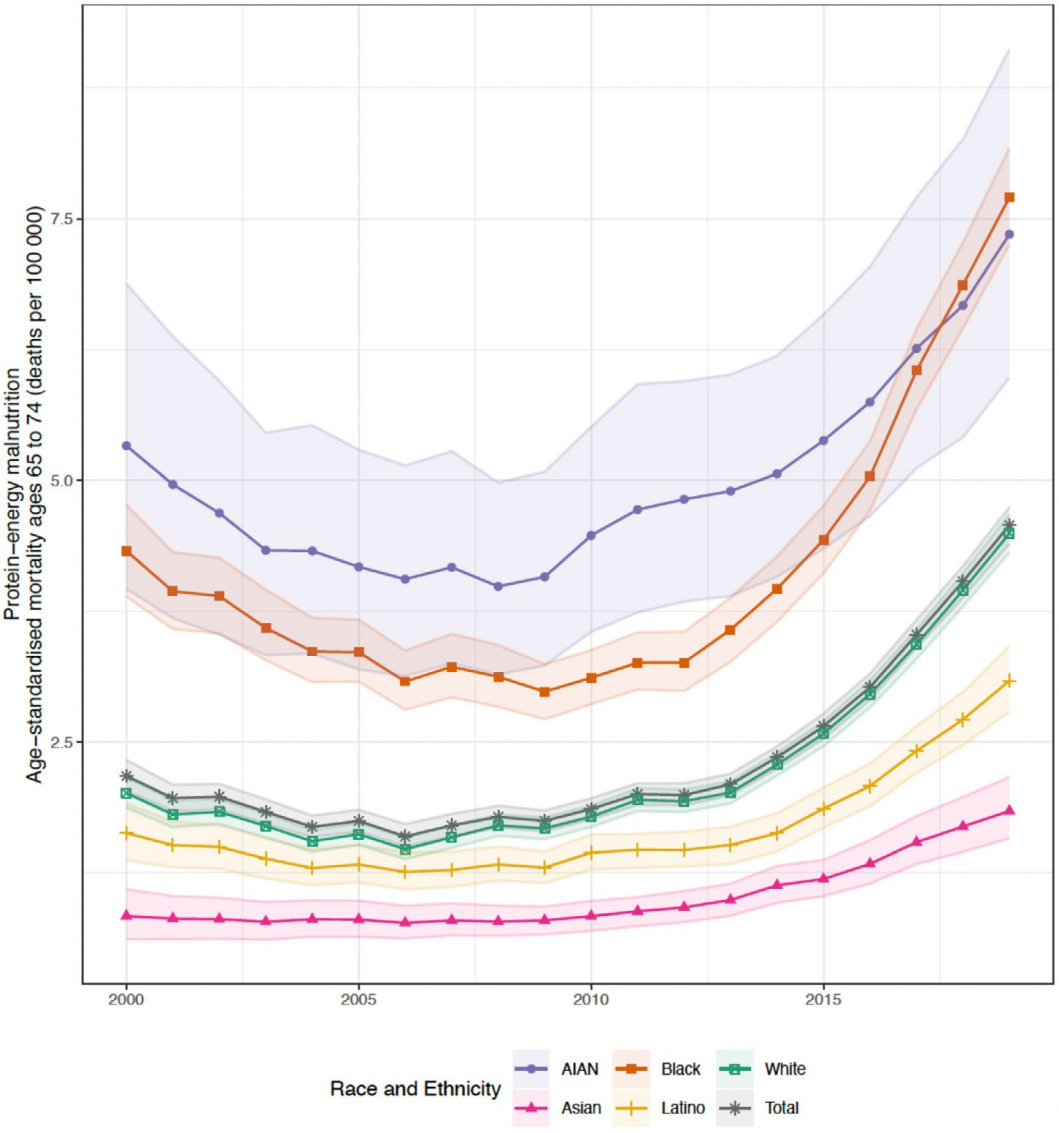
National estimated age‐standardized protein‐energy malnutrition mortality rates for individuals aged 65 to 74, by year and race and/or ethnic population groups, 2000–2019, U.S. Shaded areas are 95% uncertainty intervals.

From 2000 to 2019, malnutrition mortality rates were higher among individuals aged ≥ 75 years (2019 49.2 deaths [UI 48.4–50.0] per 100,000) than those aged 65–74 (2019 4.6 deaths [UI 4.4–4.7] per 100,000). From 2000 to 2019, mortality rates increased in both age groups, with the largest increase observed in the ≥ 75 age group since 2013 (Figure 1). Between 2000 and 2019, the absolute mortality rate in the ≥ 75 age group increased by 29.7 deaths (UI 28.6–30.8) per 100,000, and in the 65–74 age group increased by 2.4 deaths (UI 2.2–2.6) per 100,000 (Figures 1 and 2).

### 3.1 Differences in Malnutrition Mortality by Race and Ethnic Population and Age

Population differences were evident, with the highest mortality rates in 2019 being among Black (ages 65–74 years 7.7 deaths [UI 7.3–8.2] per 100,000; ≥ 75 years 60.8 deaths [UI 58.2–63.3] per 100,000) and AIAN populations (65–74 years 7.4 deaths [UI 6.0–9.1] per 100,000; ≥ 75 years 56.9 deaths [UI 47.6–66.5] per 100,000), followed by White (65–74 years 4.5 deaths [UI 4.3–4.7] per 100,000; ≥ 75 years 50.6 deaths [UI 49.7–51.4] per 100,000) and Latino (65–74 years 3.1 deaths [UI 2.8–3.4] per 100,000; ≥ 75 years 36.8 deaths [UI 34.8–38.9] per 100,000) populations. The lowest mortality rate was among Asian (65–74 years 1.8 deaths [UI 1.6–2.2] per 100,000; ≥ 75 years 24.6 deaths [UI 22.5–26.7] per 100,000) populations. Mortality rates for all groups increased between 2010 and 2019 (Figures 1 and 2).

### 3.2 County‐Level Variation in Malnutrition Mortality by Age

**FIGURE 3:**
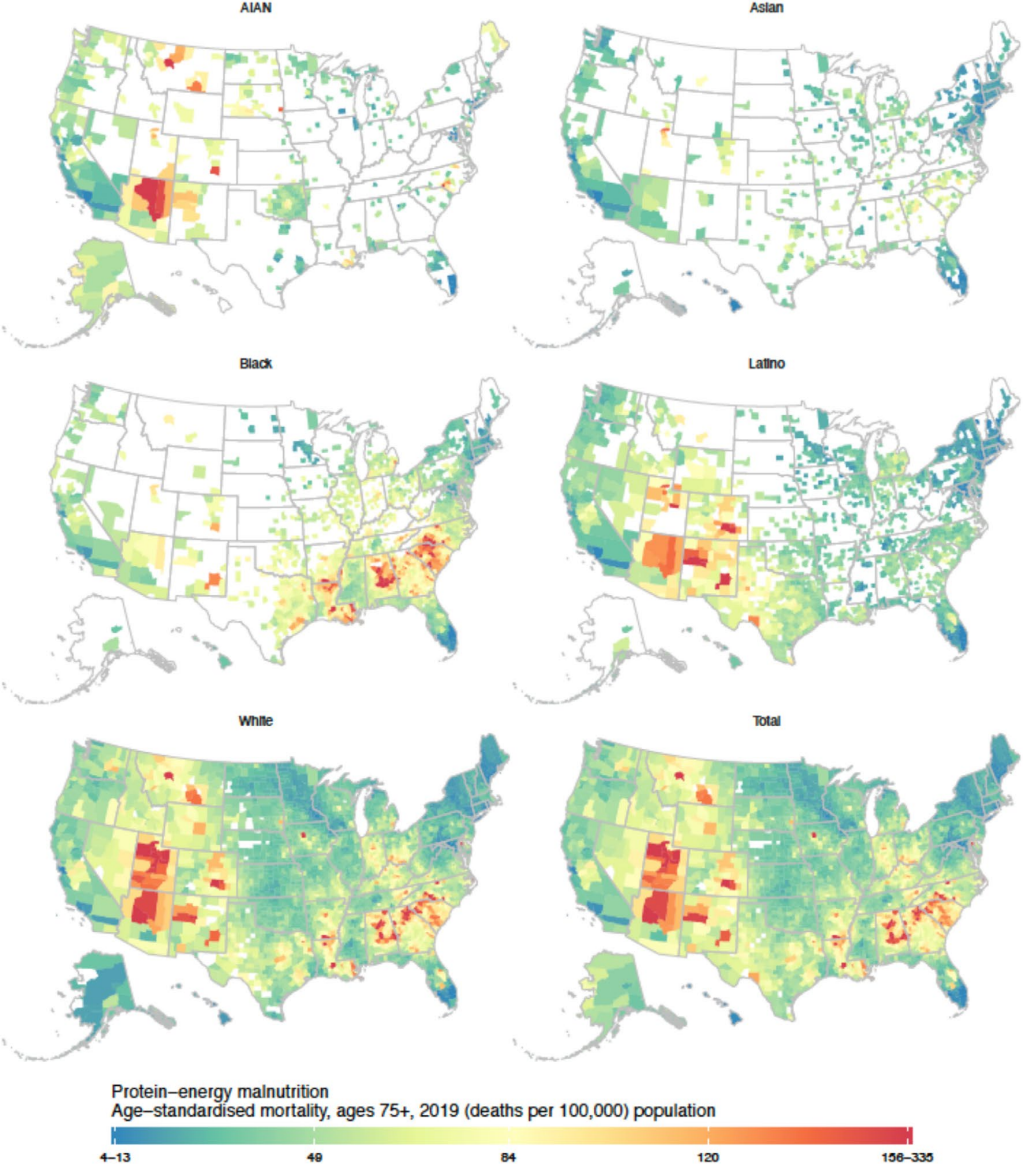
County‐level estimated age‐standardized protein‐energy malnutrition mortality rates for individuals aged ≥ 75 by race and/or ethnic population groups, 2019, U.S. Estimates are masked (shown in white) for county and race and/or ethnicity combinations with a mean annual population < 1000.

**FIGURE 4:**
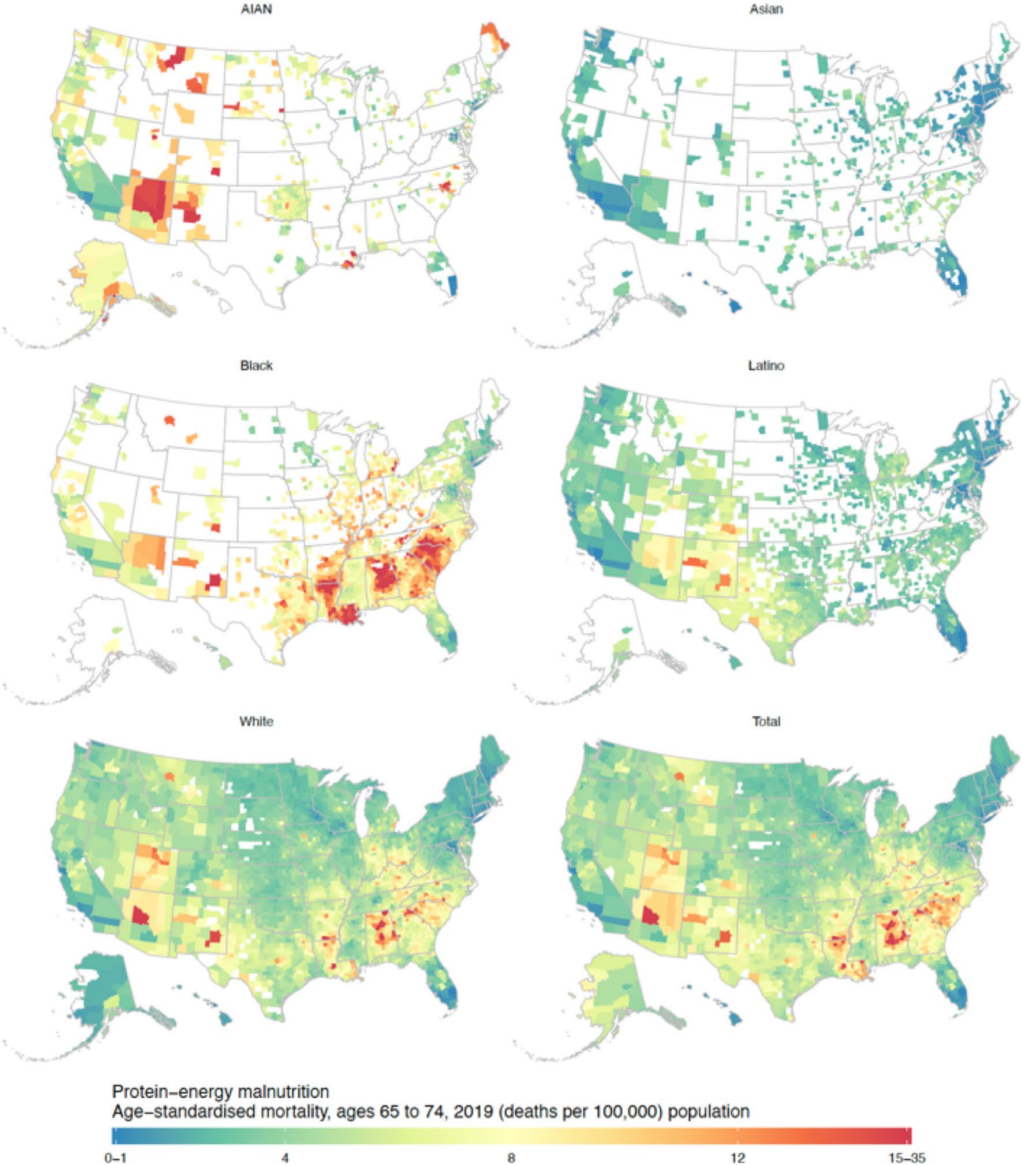
County‐level estimated age‐standardized protein‐energy malnutrition mortality rates for individuals aged 65 to 74 by race and/or ethnic population groups, 2019, U.S. Estimates are masked (shown in white) for county and race and/or ethnicity combinations with a mean annual population < 1000.

To compare counties, we highlighted those in the top 1% with the highest mortality rates for total and race‐specific population in the main text and Supporting Information, and those in the bottom 1% with the lowest mortality rates for total and race‐specific population in the main text and Supporting Information. In 2019, overall county‐level malnutrition mortality varied substantially among individuals aged ≥ 75 years (4.9 [UI 3.6–6.6] to 308.9 [UI 217.0–425.9] deaths per 100,000, median 49.3, interquartile range [IQR] 36.9–67.4; Figure 3) and those aged 65–74 (0.5 [UI 0.3–0.7] to 24.0 [UI 15.7–34.9] deaths per 100,000, median 4.9, IQR 3.7–6.6; Figure 4).

The 31 counties with the top 1% highest malnutrition mortality rate for individuals aged ≥ 75 (≥ 158.4 deaths per 100,000) were in the states of Alabama, Arizona, Arkansas, Colorado, Georgia, Iowa, Louisiana, Maryland, Montana, New Mexico, North Carolina, Utah, and Virginia. The 31 counties with the bottom 1% lowest mortality rate for individuals aged ≥ 75 (≤ 16.0 deaths per 100,000) were in California, Connecticut, Florida, Hawaii, Maine, Maryland, Minnesota, New Jersey, New York, Rhode Island, and Virginia (Figure 4).

Across all counties and the total population, the 31 counties with the top 1% highest malnutrition mortality rate for individuals aged 65–74 (≥ 13.6 deaths per 100,000) were in Alabama, Arizona, Arkansas, Georgia, Louisiana, North Carolina, South Carolina, Texas, and Virginia. The 31 counties with the bottom 1% lowest mortality rate for individuals aged 65–74 (≤ 1.5 deaths per 100,000) were in California, Connecticut, Florida, Hawaii, Maine, Maryland, Minnesota, New Jersey, New York, and Rhode Island (Figure 3).

### 3.3 Malnutrition Mortality by County, Race and Ethnicity, and Age: ≥ 75 Years Old

The top 1% highest rates for county‐level malnutrition mortality were ≥ 154.0 deaths per 100,000, while the lowest 1% mortality rates were ≤ 12.3 deaths per 100,000. The Black population experienced the highest median county‐level malnutrition mortality rate at 66.1 deaths per 100,000, followed by the White population at 48.1, AIAN at 47.8, and Latino populations at 41.1 deaths per 100,000. The Asian population had the lowest median rate, with 39.3 deaths per 100,000. There were 22 counties with mortality rates of ≥ 154.0 per 100,000 among the Black population (Table S4); 40 counties for the White population (Supplemental Table S5); 4 counties for the AIAN population (Table S6); and 6 counties for the Latino population (Table S7).

Most counties with the highest mortality rates for Black (59.1%) and White individuals (62.5%) aged ≥ 75 were located in metropolitan areas. Half of the counties with the highest mortality rates for AIAN (50%) and Latino (57.1%) individuals were situated in non‐metropolitan areas. In North Carolina, for example, counties with the highest mortality rates for Black and White individuals aged ≥ 75 were found both in the city of Charlotte and rural areas. Arizona and Montana counties presented the highest mortality rates for the AIAN and White populations in Flagstaff, Great Falls, and the rural regions. In Colorado, New Mexico, and Utah, counties showed mortality rates of 154.0 or more deaths per 100,000 for Latino and White populations aged ≥ 75 in Colorado Springs, Albuquerque, Salt Lake City, and non‐metropolitan areas (Figure 3).

There were 9 counties for the Black population (Table S4), 12 counties for the White population (Table S5), 9 counties for the AIAN population (Table S6), 23 counties for the Latino population (Table S7), and 19 counties for the Asian population (Table S8) with malnutrition mortality rates of 12.3 or fewer deaths per 100,000. Most counties with the lowest mortality rates for all population groups aged ≥ 75 were located in metropolitan areas (Figure 3).

### 3.4 Malnutrition Mortality by County, Race and Ethnicity, and Age: 65–74 Years Old

In 2019, county‐level malnutrition mortality for individuals aged 65–74 varied significantly across population groups. The highest rates, top 1%, were ≥ 15.8 deaths per 100,000, while the lowest rates, bottom 1%, were ≤ 1.1 deaths per 100,000. Among all population groups, the Black population experienced the highest median county‐level malnutrition mortality rate at 8.4 deaths per 100,000, followed by the AIAN at 6.6, White at 4.7, and Latino at 3.5 deaths per 100,000. The Asian population had the lowest median rate, with 2.7 deaths per 100,000. These counties are listed in Tables S4–S8. Most counties with the highest mortality rates for Black (69.8%) and AIAN individuals (77.8%) aged 65–74 were in metropolitan areas. In contrast, 60% of counties with the highest mortality rates for White individuals in the same age group were situated in non‐metropolitan areas.

In 2019, we identified counties with the lowest 1% mortality rates, defined as 1.1 or fewer deaths per 100,000. Specifically, there were 4 counties for the Black population (Table S4), 4 counties for the AIAN population (Table S6), 10 counties for the White population (Supplemental Table S5), 25 counties for the Latino population (Table S7), and 29 counties for the Asian population (Table S8).

## 4 Discussion

This study presents the first comprehensive analysis of malnutrition mortality trends in older adults across the United States by county and race and ethnic population groups. Nationally, the substantial increase in malnutrition mortality since 2013 is concerning. These analyses showed much higher mortality rates among individuals aged 75 and older, as may be expected, but with striking regional differences. Certain southern states have usually been found to have the worst outcomes, and this was also observed for the counties in the top 1% of malnutrition mortality for Black and White populations. In addition, counties in western states had the highest malnutrition mortality for AIAN and Latino populations. These findings underscore the need for further research to understand why these mortality rates have increased since 2013 and highlight the marked differences by county and race and ethnicity groups within the same age group.

The observation that malnutrition mortality is higher for persons aged ≥ 75 years (49.2 deaths per 100,000) compared to those aged 65–74 (4.6 deaths per 100,000) may be explained by aging, lower dietary intake of energy and nutrients [29], more conditions associated with nutrition deficit, especially chronic obstructive pulmonary disease and dementia [30, 31], and an increase in all‐cause mortality [20]. Previous studies using national data in the United States have also reported increased malnutrition mortality among adults aged 65 and older between 2013 and 2020 [17, 18, 32].

Aging may contribute to malnutrition by altering nutrient‐sensing pathways, which are crucial for maintaining cellular homeostasis. As an individual ages, pathways such as mitochondrial activity, DNA damage response, telomere maintenance, and autophagy become less efficient. These pathways are integral not only to the aging process but also to the development of various diseases. The decline in cellular homeostasis efficiency with age can lead to impaired nutrient absorption, metabolism, and utilization, thereby increasing susceptibility to malnutrition [5].

The Patient Protection and Affordable Care Act led to a reclassification of malnutrition care from a non‐complicating condition to a complicating condition, allowing hospitals to receive reimbursement for treating malnutrition and improving care delivery [33]. This coding change was fully implemented by 2015 [34]. A sharp increase was observed despite the expectation that facilitating hospital reimbursements for malnutrition care would decrease mortality. One possible explanation is that recognizing malnutrition as a complicating condition may have improved sensitivity to patient illness severity, leading to increased diagnosis of a previously undiagnosed condition, and incentivized by the option to bill. Alternatively, the change in reimbursement policy might have incentivized overdiagnosis due to the higher Medicare reimbursement rates [34]. Regardless of whether malnutrition was previously underdiagnosed or subsequently overdiagnosed, the magnitude of the mortality increase suggests that other factors, possibly related to social determinants of health rather than clinical care, may also play a significant role.

Reporting malnutrition as the primary cause of death on a death certificate may also reflect food insecurity and inadequate access to healthcare [35]. Food insecurity may force individuals to make difficult financial decisions, such as buying food or paying for medical expenses, leading to delayed or deferred medical care [35]. Among older adults, food insecurity more than doubled between 2007 and 2016 and continued to rise through 2019 [36]. In 2019, counties like Scotland County, North Carolina; Chilton County, Alabama; and Cibola County, New Mexico, experienced both higher malnutrition mortality rates and food insecurity prevalence of 19.7%, 17.2%, and 19.6%, respectively [37]. In contrast, counties with lower malnutrition mortality rates, such as Queens County, New York; Palm Beach County, Florida; and Hudson County, New Jersey, had lower food insecurity prevalence rates of 9.7%, 10.6%, and 11% [37]. Interestingly, the majority of the counties with lower malnutrition mortality rates set a higher poverty level threshold for eligibility to food assistance programs, like the Supplemental Nutritional Assistance Program (SNAP), ranging from 185% to 200% of the federal poverty level [37]. Meanwhile, counties with higher malnutrition mortality rates had a lower poverty level threshold for SNAP eligibility, ranging from 130% to 165% [37]. Enhancing nutritional programs, expanding community‐based health and nutrition education, and improving access to healthcare for screening and management are potential components of targeted interventions that may reduce mortality.

Routine malnutrition screening for older adults is not standard in hospitals or outpatient clinical settings and may only be suggested for those underweight [4, 38, 39]. The lack of screening may contribute to higher mortality rates due to underdiagnosis or misdiagnosis [40]. Malnutrition deaths occur often in hospital settings (37%) [17], highlighting the need for nutritional support in clinical settings to reduce differences and enhance healthy aging [41]. Obstacles to healthcare access in these communities further delay diagnosis and treatment, impeding timely nutritional and clinical interventions [17, 40]. This highlights the need for nutritional support in clinical settings to reduce differences and enhance healthy aging [41]. Additionally, nutrition education for older adults and caregivers could sustain nutritional improvements. Public and private programs, such as Meals on Wheels, have successfully reduced hospitalizations and hospital length of stay related to malnutrition [42, 43]. Tailoring these programs with strategies to prevent and treat malnutrition, adequate funding, and simplified enrollment could further reduce malnutrition in older adults.

The elevated mortality rates among Black populations in metropolitan areas highlight heightened concerns compared to most other populations [18]. A prior study focused on older adults in healthcare settings identified that Black adults faced the highest nutritional risk among population groups, with nearly 21% reporting consumption of fewer than two meals per day. In comparison, only 2% of White and Asian adults reported consuming fewer than two meals daily [40]. Similarly, the high mortality rates observed among AIAN populations highlight that they have the second‐highest malnutrition mortality rate [17]. A qualitative study involving AIAN older adults in urban areas underscored that, although food assistance programs exist, they may not be adequate, given that the food provided is often high in carbohydrates and limited in fresh produce or protein‐rich options. Transportation barriers, difficulty carrying groceries, and cultural and historical factors are all potential obstacles to healthy eating for older AIAN adults [44]. Finally, the higher mortality rates in counties in rural areas of the South and Southwest were notable and add to the evidence of the rural–urban divergence in health outcomes [45, 46].

Because malnutrition mortality for Asian and Latino older adults was lower than the national average, there may be protective factors that collectively enhance the resilience of these populations against malnutrition. Asian and Latino older adults are more likely to live in multigenerational households, which may provide more family caregiver support [47, 48]. National data also indicate that Asian and Latino adults generally have a higher average protein consumption compared to Black, White, and other population groups [49]. However, further research is needed to explore the dietary patterns of older Latino and Asian adults that may contribute to protection against malnutrition later in life. A study conducted in New York City found that a supportive and cohesive social network benefited the Latino population with higher diet quality and food security [50]. This social support and cohesion may be linked to the resilience of the older Latino population in facing malnutrition.

The limitations of this systematic approach to evaluate population health were previously described [19] and include potential inaccuracies in death, population, and covariate data, as well as limitations in methods used to correct racial and/or ethnic misclassification and validate small‐area estimation models. The uncertainty intervals do not capture all sources of uncertainty, such as measurement and model biases. The combined Asian and NHPI grouping may obscure within‐population differences, with estimates primarily reflecting outcomes for Asians due to their larger population size.

In conclusion, our study highlights the critical need for targeted clinical, public health, and policy interventions to reduce malnutrition mortality among older adults in the US. The population differences in mortality rates and the wide variation by county underscore the necessity for culturally and regionally tailored nutrition programs that address the specific socioeconomic and environmental challenges these groups face. Refining strategies for screening, detection, evaluation, and provision of nutritional support within healthcare and community settings may significantly reduce mortality rates associated with malnutrition, especially in rural areas. Engaging communities meaningfully and expanding community‐based nutrition education and support programs may help mitigate many underlying causes of malnutrition. Our findings emphasize the importance of further research to identify protective factors in all populations, which could inform the development of resilience‐building strategies to empower communities to thrive.

## Author Contributions

All authors had full access to all of the study's data and are responsible for the integrity and accuracy of the data analysis. Concept and design: J.T.C., E.J.R., A.S.H., and E.J.P.‐S. Acquisition, analysis, or interpretation of data: J.T.C., J.O.M., E.J.R., A.S.H., L.D.‐L., A.H.M., and E.J.P.‐S. Drafting of the manuscript: J.T.C. and J.O.M. Critical review of the manuscript for important intellectual content: All authors. Statistical analysis: L.D.‐L. Obtained funding: E.J.P.‐S. Administrative, technical, or material support: E.J.R., A.S.H, and C.S.A. Supervision: E.J.R., A.S.H., and E.J.P.‐S.

## Conflicts of Interest

The authors declare no conflicts of interest.

## Disclosure

This research was supported [in part] by the Intramural Research Program of the National Institutes of Health (NIH). The contributions of the NIH author(s) are considered Works of the United States Government. The findings and conclusions presented in this paper are those of the author(s) and do not necessarily reflect the views of the NIH or the U.S. Department of Health and Human Services.

## Supporting information


**Data S1:** jgs70042‐sup‐0001‐Supinfo.pdf.

## Data Availability

Upon publication of this article, all county and population estimates can be accessed through an online visualization tool https://vizhub.healthdata.org/subnational/usa and via the Global Health Data Exchange (https://ghdx.healthdata.org/us‐data).
