# Supplementary material for: Malnutrition Mortality Among Older Adults by County and Race and/or Ethnicity in the United States, 2000–2019
Source: J Am Geriatr Soc. 2025 Sep 12;73(9):2868–77. doi: 10.1111/jgs.70042 (PMC12460942; doi:10.1111/jgs.70042)
Supplement: Supplementary file 1 — Data S1: jgs70042‐sup‐0001‐Supinfo.pdf. [file JGS-73-2868-s001.pdf]

SUPPLEMENTAL FIGURE S1. National estimated age-standardized protein-energy malnutrition mortality rates for individuals aged 55 to 64, by year and race and/or ethnic population groups, 2000-2019, U.S.
Shaded areas are 95% uncertainty intervals.

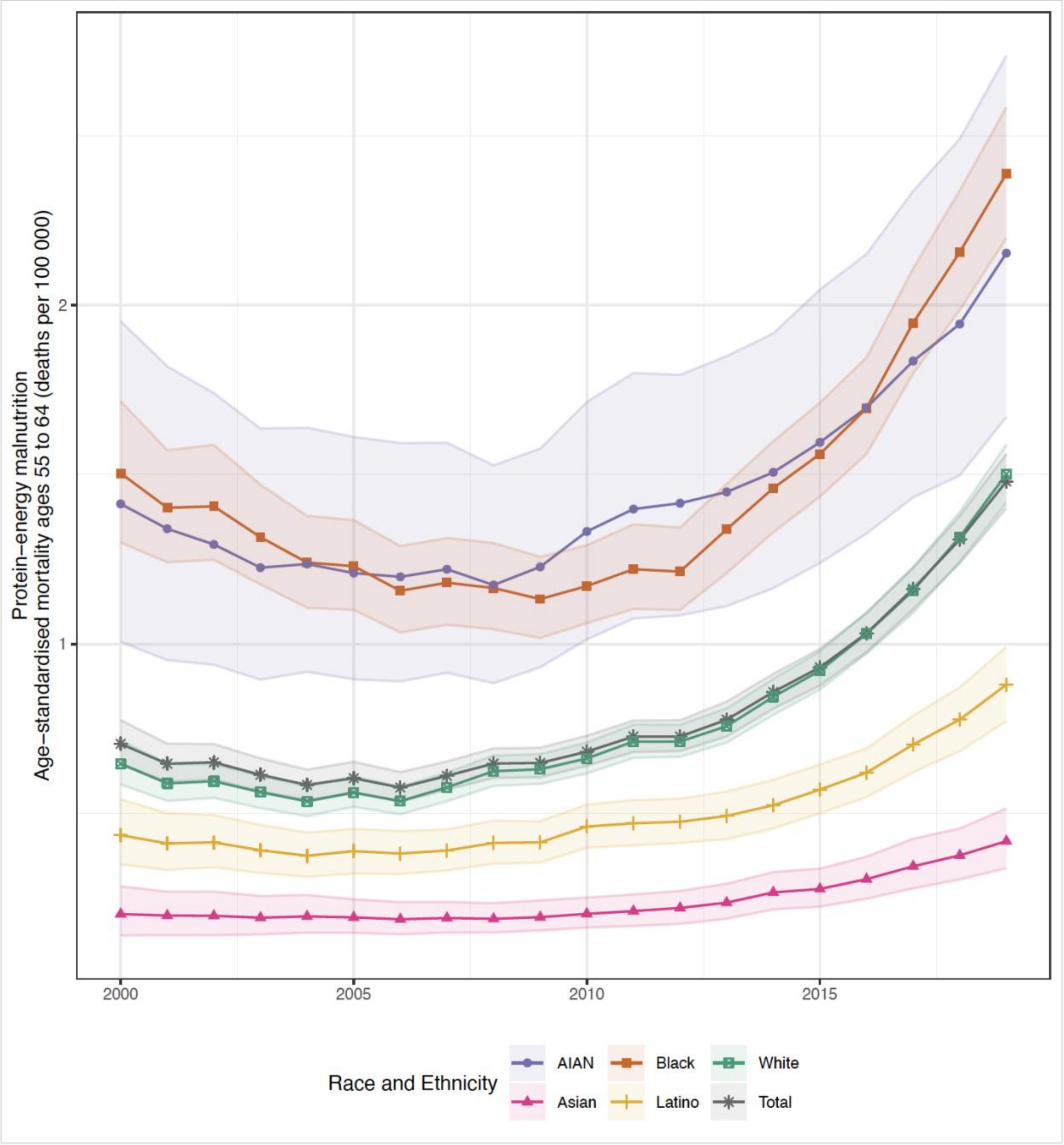

SUPPLEMENTAL FIGURE S2. County-level estimated age-standardized protein-energy malnutrition mortality rates for individuals aged 55 to 64 by race and/or ethnic population groups, 2019, U.S.

Estimates are masked (shown in white) for county and race and/or ethnicity combinations with a mean annual population <1000.

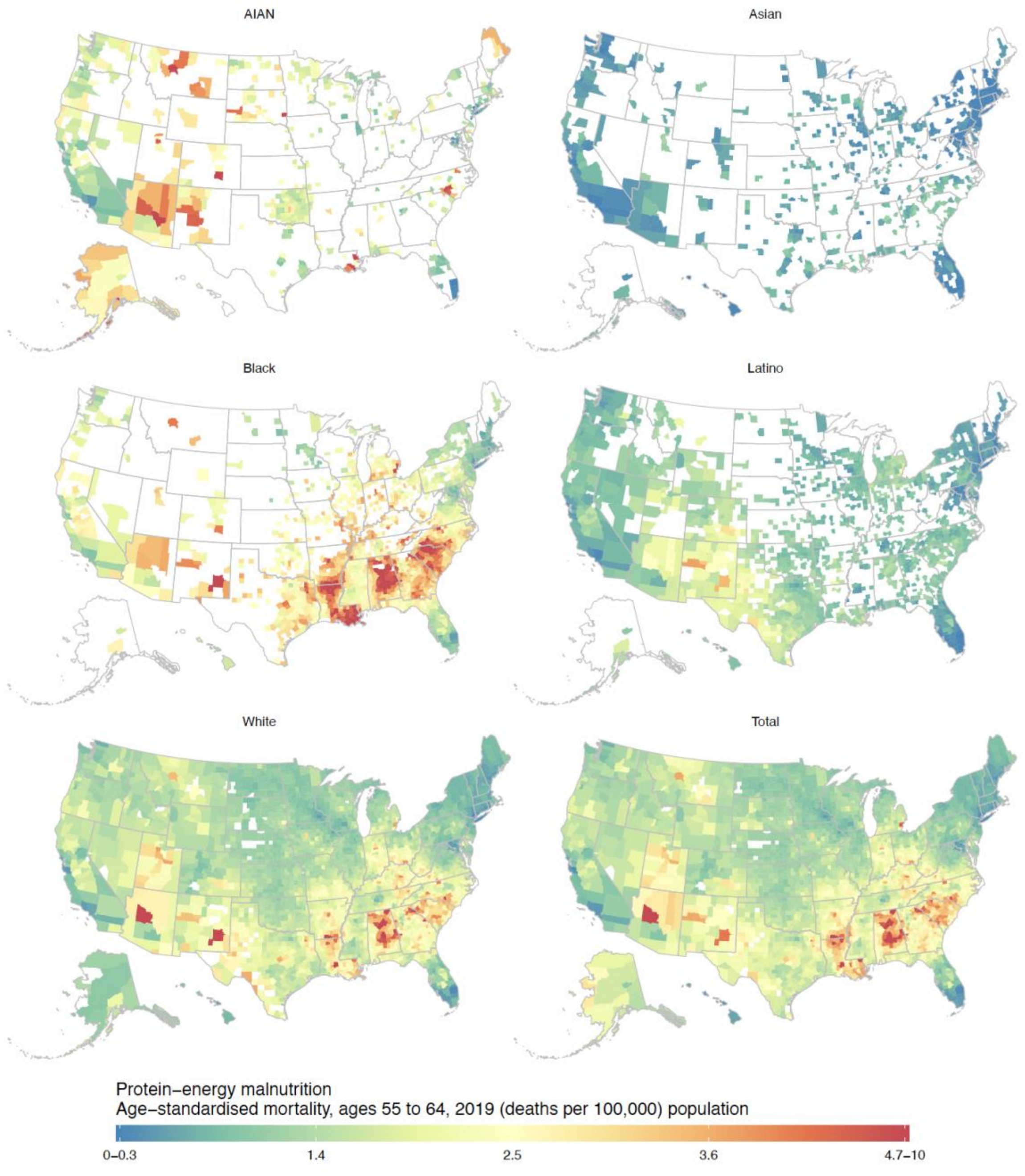

SUPPLEMENTAL TABLE S3. GATHER checklist

**Item #**	**Checklist item**	**Description of Compliance**
Objectives and funding		
1	Define the indicator(s), populations (including age, sex, and geographic entities), and time period(s) for which estimates were made.	Methods section
2	List the funding sources for the work.	Funding/Support section
Data Inputs		
For all data inputs from multiple sources that are synthesized as part of the study:		
3	Describe how the data were identified and how the data were accessed.	Methods section
4	Specify the inclusion and exclusion criteria. Identify all ad-hoc exclusions.	Methods section
5	Provide information on all included data sources and their main characteristics. For each data source used, report reference information or contact name/institution, population represented, data collection method, year(s) of data collection, sex and age range, diagnostic criteria or measurement method, and sample size, as relevant.	See Dwyer-Lindgren et al. 2023 (citation #19 in main text)
6	Identify and describe any categories of input data that have potentially important biases (eg based on characteristics listed in item 5).	Limitations section
For data inputs that contribute to the analysis but were not synthesized as part of the study:		
7	Describe and give sources for any other data inputs.	N/A
For all data inputs:		
8	Provide all data inputs in a file format from which data can be efficiently extracted (eg a spreadsheet rather than a PDF), including all relevant meta-data listed in item 5. For any data inputs that cannot be shared because of ethical or legal reasons, such as third-party ownership, provide a contact name or the name of the institution that retains the right to the data.	GHDx (to be added upon publication)
Data analysis		
9	Provide a conceptual overview of the data analysis method. A diagram may be helpful.	Methods section

10	Provide a detailed description of all steps of the analysis, including mathematical formulae. This description should cover, as relevant, data cleaning, data pre-processing, data adjustments and weighting of data sources, and mathematical or statistical model(s).	Methods section, further details in Dwyer-Lindgren et al. 2023 (citation #19 in main text)
11	Describe how candidate models were evaluated and how the final model(s) were selected.	See Dwyer-Lindgren et al. 2023 (citation #19 in main text)
12	Provide the results of an evaluation of model performance, if done, as well as the results of any relevant sensitivity analysis.	N/A
13	Describe methods for calculating uncertainty of the estimates. State which sources of uncertainty were, and were not, accounted for in the uncertainty analysis.	Methods section
14	State how analytic or statistical source code used to generate estimates can be accessed.	GitHub
Results and Discussion		
15	Provide published estimates in a file format from which data can be efficiently extracted.	GHDx (to be added upon publication)
16	Report a quantitative measure of the uncertainty of the estimates (e.g. uncertainty intervals).	Results section, GHDx (to be added upon publication)
17	Interpret results in light of existing evidence. If updating a previous set of estimates, describe the reasons for changes in estimates.	Discussion section
18	Discuss limitations of the estimates. Include a discussion of any modelling assumptions or data limitations that affect interpretation of the estimates.	Discussion section

SUPPLEMENTAL TABLE S4. Counties with the Highest and Lowest Protein-Energy Malnutrition Mortality for the Black Population by Age Group in 2019

State	County	Rural Urban Classification	Rural-Urban Continuous Code	Main cities	Population (n)	Mortality (deaths per 100,000)	Uncertainty Interval
≥ 75 Highest Mortality							
South Carolina	Cherokee	Non-metro	6	Gaffney	510	208.3	99.0 – 368.7
Arkansas	Union	Non-metro	7	El Dorado	609	205.8	122.9 – 312.8
North Carolina	Scotland	Non-metro	6	Laurinburg	718	196.2	115.5 – 298.2
Alabama	Lee	Metro	3	Opelika and Auburn	1,229	184.1	111.6 – 278.0
Alabama	Jefferson	Metro	1	Birmingham	13,063	183.4	143.6 – 227.2
Louisiana	Saint Landry Parish	Non-metro	4	Opelousas	1,639	176.0	113.8 – 254.1
Alabama	Autauga	Metro	2	Prattville and Montgomery	462	171.7	92.4 – 285.5
Alabama	Chilton	Metro	1	Clanton, Birmingham and Montgomery	206	168.9	84.1 – 284.7
Alabama	Montgomery	Metro	2	Montgomery	5,381	168.8	124.7 – 221.1
Alabama	Lowndes	Metro	2	Hayneville	491	166.5	99.2 – 259.5
South Carolina	York	Metro	1	Rock Hill and Charlotte	1,672	165.9	103.0 – 247.5
South Carolina	Allendale	Non-metro	8	Allendale	401	165.7	87.8 – 271.7
Alabama	Macon	Metro	3	Tuskegee	1,118	164.4	108.0 – 233.7
Alabama	Dallas	Non-metro	4	Selma	1,474	163.0	97.7 – 241.4
North Carolina	Mecklenburg	Metro	1	Charlotte	10,781	162.7	123.3 – 202.9
Georgia	Muscogee	Metro	2	Columbus	3,868	158.6	108.0 – 217.2
North Carolina	Catawba	Metro	2	Newton and Hickory	610	156.7	83.6 – 262.8
North Carolina	Union	Metro	1	Monroe and Charlotte	1,127	156.3	92.8 – 239.3
South Carolina	Richland	Metro	2	Columbia	6,900	155.4	115.7 – 202.8
South Carolina	Barnwell	Non-metro	8	Barnwell	480	155.4	78.0 – 270.7
South Carolina	Greenwood	Non-metro	4	Greenwood	1,036	154.3	89.6 – 237.8
Virginia	Martinsville	Non-metro	4	Martinsville	255	154.1	34.6 – 392.7
≥ 75 Lowest Mortality							
Florida	Palm Beach	Metro	1	Jupiter, Palm Beach Gardens, Wellington, and West Palm Beach	11,107	11.9	6.6 – 19.7
New York	Westchester	Metro	1	New Rochelle, Scarsdale, Tarrytown, White Plains, and Yonkers	8,804	11.0	5.7 – 18.8
Florida	Martin	Metro	2	Palm city, Stuart	386	10.7	4.8 – 19.6
New York	Richmond	Metro	1	New York - Staten Island	1,872	8.7	3.9 – 17.4

New York	New York	Metro	1	New York - Manhattan	15,822	8.0	4.3 – 13.6
New York	Nassau	Metro	1	Hempstead	9,695	7.3	3.5 – 13.2
New York	Bronx	Metro	1	New York - Bronx	24,132	5.6	3.2 – 9.4
New York	Queens	Metro	1	New York - Queens	28,710	5.3	3.0 – 8.5
New York	Kings	Metro	1	New York - Brooklyn	51,485	4.4	2.3 – 7.2
65-74 Highest Mortality							
North Carolina	Scotland	Non-metro	6	Laurinburg	1,388	27.9	15.8 – 43.5
Arkansas	Union	Non-metro	7	El Dorado	1,091	25.8	15.3 – 40.0
Louisiana	Saint Landry Parish	Non-metro	4	Opelousas	2,674	24.9	14.8 – 37.9
Louisiana	Lafourche Parish	Metro	3	Thibodaux	852	23.7	12.3 – 39.3
South Carolina	Cherokee	Non-metro	6	Gaffney	896	23.6	12.1 – 39.8
Alabama	Jefferson	Metro	1	Birmingham	25,042	22.0	16.3 – 28.8
Louisiana	Washington Parish	Non-metro	6	Franklinton and Bogalusa	1,293	21.9	11.9 – 34.4
Louisiana	Saint Tammany Parish	Metro	2	Covington, Slidell, and New Orleans	2,242	21.4	12.9 – 34.0
Alabama	Autauga	Metro	2	Prattville and Montgomery	800	19.9	9.4 – 33.3
Alabama	Chambers	Non-metro	6	Lafayette and Valley	1,275	19.8	10.3 – 32.4
Virginia	Radford City	Metro	3	Radford	69	19.7	4.4 – 51.3
Louisiana	Saint Bernard Parish	Metro	1	Chalmette and New Orleans	482	19.4	8.7 – 36.4
Louisiana	Lincoln Parish	Non-metro	4	Ruston	1,168	19.4	9.8 – 31.4
Louisiana	East Baton Rouge Parish	Metro	2	Baton Rouge	14,529	18.9	13.2 – 26.0
Alabama	Chilton	Metro	1	Clanton, Birmingham and Montgomery	369	18.7	9.0 – 33.4
South Carolina	Lancaster	Metro	1	Lancaster	1,768	18.7	10.9 – 29.7
Texas	Camp	Non-metro	8	Pittsburg	248	18.6	7.6 – 35.2
Alabama	Montgomery	Metro	2	Montgomery	9,857	18.4	13.1 – 24.8
Alabama	Lowndes	Metro	2	Hayneville	695	18.3	10.5 – 29.0
Louisiana	Jefferson	Metro	1	Gretna and New Orleans	8,626	18.2	12.1 – 26.5
Georgia	Stephens	Non-metro	7	Toccoa	304	18.2	7.2 – 36.8
Louisiana	Ouachita	Metro	3	Monroe	3,773	18.0	10.4 – 27.6
Alabama	Lee	Metro	3	Opelika and Auburn	2,681	17.8	9.8 – 29.3
Louisiana	Acadia Parish	Metro	2	Crowley and Lafayette	774	17.8	9.0 – 32.1
Georgia	Habersham	Non-metro	6	Clarkesville	89	17.6	7.7 – 36.0
South Carolina	York	Metro	1	Rock Hill and Charlotte	3,829	17.5	10.6 – 27.0
Alabama	Dallas	Non-metro	4	Selma	2,481	17.4	10.0 – 26.5
South Carolina	Chester	Metro	1	Chester and Charlotte	1,114	17.3	8.9 – 28.3
North Carolina	Catawba	Metro	2	Newton and Hickory	1,204	17.2	8.2 – 30.5

Louisiana	Terrebonne	Metro	3	Houma and New Orleans	1,569	17.1	8.0 – 31.6
Alabama	Elmore	Metro	2	Wetumpka and Montgomery	950	17.0	9.1 – 28.4
North Carolina	Mecklenburg	Metro	1	Charlotte	21,946	17.0	12.8 – 22.3
Louisiana	Morehouse Parish	Metro	3	Bastrop and Monroe	1,019	17.0	8.8 – 27.0
Louisiana	West Carroll Parish	Non-metro	8	Oak Grove	142	16.9	6.5 – 36.4
South Carolina	Anderson	Metro	2	Anderson and Greenville	2,649	16.8	9.8 – 26.0
South Carolina	Spartanburg	Metro	2	Spartanburg and Greenville	4,955	16.6	10.1 – 24.8
Texas	Gregg	Metro	2	Longview and Tyler	1,906	16.6	8.5 – 28.1
Michigan	Wayne	Metro	1	Detroit	59,370	16.6	12.7 – 21.2
Louisiana	Jackson Parish	Non-metro	6	Jonesboro	402	16.6	7.0 – 31.5
South Carolina	Fairfield	Metro	2	Winnsboro and Columbia	1,346	16.6	9.7 – 25.7
Alabama	Macon	Metro	3	Tuskegee	1,809	16.4	9.6 – 24.9
South Carolina	Barnwell	Non-metro	8	Barnwell	918	16.3	8.6 – 27.8
South Carolina	Union	Metro	2	Union and Spartanburg	942	16.3	9.2 – 26.8
Georgia	Haralson	Metro	1	Buchanan and Atlanta	128	16.2	6.1 – 35.6
Louisiana	Plaquemines	Metro	1	Pointe à la Hache and New Orleans	349	16.2	6.5 – 32.5
Virginia	Martinsville City	Non-metro	4	Martinsville	538	16.1	4.1 – 42.1
North Carolina	Cleveland	Non-metro	4	Shelby	1,951	16.0	9.0 – 26.1
North Carolina	Lincoln	Metro	1	Lincolnton and Charlotte	466	16.0	8.1 – 28.9
Louisiana	Saint James Parish	Metro	1	Baton Rouge and New Orleans	1,065	15.9	8.2 – 26.7
North Carolina	Gaston	Metro	1	Charlotte	2,741	15.9	8.1 – 25.3
North Carolina	Rockingham	Metro	2	Eden and Greensboro	2,014	15.9	8.8 – 25.5
Alabama	Saint Clair	Metro	1	Birmingham	547	15.8	7.3 – 29.6
Alabama	Etowah	Metro	3	Gadsden	1,438	15.8	8.1 – 28.0
65-74 Lowest Mortality							
New York	Nassau	Metro	1	Hempstead	12,868	0.9	0.4 – 1.6
New York	Bronx	Metro	1	New York - Bronx	33,106	0.8	0.4 – 1.3
New York	Queens	Metro	1	New York - Queens	38,612	0.7	0.4 – 1.2
New York	Kings	Metro	1	New York - Brooklyn	72,181	0.6	0.3 – 0.9

The highest protein-energy malnutrition mortality thresholds were chosen based on the 99th percentile of mortality for each age group, and lowest based on the 1st percentile of mortality for each age group. For the age group ≥ 75 years old, the highest (99th) threshold was ≥ 154.0 per 100,000 and the lowest (1st) threshold was ≤ 12.3. For the age group 65-74 years old, the highest (99th) threshold was ≥ 15.8 per 100,000 deaths and the lowest (1st) was ≤ 1.1.

Rural-Urban continuous code: 1 - counties in metro areas of 1 million population or more; 2 – counties in metro areas of 250,000 to 1 million population; 3 – counties in metro areas fewer than 250,000 population; 4 - urban population of 20,000 or more, adjacent to a metro area; 6 - urban population of 5,000 to 20,000 adjacent to a metro area; 7 - urban population of 5,000 to 20,000 not adjacent to a metro area; 8 - urban population of fewer than 5,000, adjacent to a metro area; Population: Estimated population for the age bracket, race/ethnicity, and county.

SUPPLEMENTAL TABLE S5. Counties with the Highest and Lowest Protein-Energy Malnutrition Mortality for the White Population by Age Group in 2019

State	County	Rural Urban Classification	Rural-Urban Continuous Code	Main cities	Population (n)	Mortality (deaths per 100,000)	Uncertainty Interval
≥ 75 Highest Mortality							
Georgia	Stephens	Non-metro	7	Toccoa	1,784	334.9	236.6 – 464.8
Georgia	Habersham	Non-metro	6	Clarkesville	3,342	305.6	228.5 – 392.4
Arizona	Yavapai	Metro	3	Prescott	26,187	261.8	228.0 – 296.1
Virginia	Martinsville City	Non-metro	4	Martinsville	649	258.3	95.2 – 499.6
Georgia	Muscogee	Metro	2	Columbus	6,294	241.0	187.3 – 304.0
Arkansas	Union	Non-metro	7	El Dorado	2,194	222.6	157.0 – 305.5
Georgia	Hall	Metro	3	Gainesville	11,272	209.1	171.3 – 250.6
Utah	Salt Lake	Metro	1	Salt Lake City	43,266	205.9	180.7 – 232.5
Utah	Carbon	Non-metro	7	Price	1,237	201.7	124.6 – 305.7
Utah	Utah	Metro	2	Provo and Salt Lake City	19,014	200.1	166.6 – 236.3
Utah	Tooele	Metro	1	Tooele and Salt Lake City	2,170	196.8	142.5 – 261.7
Iowa	Webster	Non-metro	5	Fort Dodge	2,916	195.0	137.1 – 269.4
North Carolina	Mecklenburg	Metro	1	Charlotte	33,610	194.5	168.4 – 220.1
Alabama	Lee	Metro	3	Opelika and Auburn	6,033	192.9	149.1 – 242.8
Virginia	Radford City	Metro	3	Radford	633	189.4	98.7 – 325.9
Alabama	Jefferson	Metro	1	Birmingham	28,439	188.6	161.3 – 216.5
Montana	Cascade	Metro	3	Great Falls	6,525	186.4	141.7 – 235.2
Georgia	Banks	Non-metro	8	Homer	1,244	180.3	119.8 – 260.5
Colorado	Pueblo	Metro	3	Pueblo and Colorado Springs	9,321	177.6	142.1 – 219.3
North Carolina	Catawba	Metro	2	Newton and Hickory	10,512	177.2	134.9 – 224.1
Georgia	Haralson	Metro	1	Buchanan and Atlanta	1,832	175.7	111.4 – 255.9
Georgia	Lumpkin	Metro	1	Dahlonega and Atlanta	2,170	175.0	119.0 – 245.7
Maryland	Queen Anne's	Metro	1	Centreville, Baltimore and Washington DC	3,577	173.9	124.1 – 239.5
Alabama	Russell	Metro	2	Phenix and Columbus	2,109	171.9	115.2 – 244.1
Utah	Duchesne	Non-metro	7	Duchesne	979	170.3	105.2 – 253.4
Alabama	Chilton	Metro	1	Clanton, Birmingham and Montgomery	2,687	169.6	117.8 – 236.6
North Carolina	Scotland	Non-metro	6	Laurinburg	1,677	168.8	114.8 – 233.4
Georgia	Walton	Metro	1	Monroe and Atlanta	5,086	164.9	121.5 – 215.5

Utah	Davis	Metro	2	Farmington and Salt Lake City	13,334	164.0	129.0 – 202.5
North Carolina	Dare	Non-metro	4	Manteo	2,657	163.0	92.3 – 249.5
Georgia	Franklin	Non-metro	8	Carnesville	1,671	162.4	105.1 – 233.3
Utah	Weber	Metro	2	Odgen and Salt Lake City	10,695	161.4	124.2 – 202.1
North Carolina	Rockingham	Metro	2	Eden and Greensboro	6,419	160.6	121.9 – 206.3
Alabama	Blount	Metro	1	Oneonta and Birmingham	4,129	160.5	114.3 – 221.6
Alabama	Autauga	Metro	2	Prattville and Montgomery	3,219	160.0	107.8 – 219.7
Louisiana	Lincoln Parish	Non-metro	4	Ruston	1,810	159.3	106.6 – 226.8
Louisiana	Saint Landry Parish	Non-metro	4	Opelousas	3,495	159.3	109.6 – 219.4
Utah	Sanpete	Non-metro	6	Manti	1,712	158.4	102.9 – 223.6
New Mexico	Bernalillo	Metro	2	Albuquerque	28,300	157.2	132.1 – 185.2
Georgia	Jackson	Non-metro	4	Jefferson	3,766	155.6	110.7 – 210.9
≥ 75 Lowest Mortality							
Rhode Island	Washington	Metro	1	Narragansett, Westerly	10,488	12.2	6.5 – 20.7
Maine	Cumberland	Metro	2	Portland	22,047	12.0	7.4 – 18.2
New York	Suffolk	Metro	1	Brookhaven	96,269	11.7	8.6 – 15.2
Florida	Lee	Metro	2	Cape Coral	83,790	10.1	6.9 – 13.8
New York	Westchester	Metro	1	New Rochelle, Scarsdale, Tarrytown, White Plains, and Yonkers	57,447	10.1	7.0 – 13.9
New York	New York	Metro	1	New York - Manhattan	66,032	8.8	6.2 – 12.2
Florida	Palm Beach	Metro	1	Jupiter, Palm Beach Gardens, Wellington, and West Palm Beach	139,747	8.3	6.3 – 11.1
New York	Richmond	Metro	1	New York - Staten Island	25,404	7.9	5.1 – 12.3
New York	Bronx	Metro	1	New York - Bronx	19,369	7.5	4.3 – 12.0
New York	Nassau	Metro	1	Hempstead	84,978	6.4	4.5 – 8.7
New York	Kings	Metro	1	New York - Brooklyn	67,258	5.4	3.5 – 8.0
New York	Queens	Metro	1	New York - Queens	66,788	5.2	3.4 – 7.6
65 – 74 Highest Mortality							
Georgia	Stephens	Non-metro	7	Toccoa	2,738	25.0	16.2 – 36.1
Georgia	Habersham	Non-metro	6	Clarkesville	4,583	21.1	15.2 – 28.7
Arkansas	Union	Non-metro	7	El Dorado	2,916	18.7	12.2 – 27.0

Virginia	Radford City	Metro	3	Radford	891	18.6	8.2 – 35.8
North Carolina	Scotland	Non-metro	6	Laurinburg	2,000	18.6	11.2 – 28.3
Arizona	Yavapai	Metro	3	Prescott	40,023	18.2	14.4 – 22.1
Alabama	Chilton	Metro	1	Clanton, Birmingham and Montgomery	4,055	16.5	10.8 – 23.7
Virginia	Martinsville	Non-metro	4	Martinsville	739	16.3	5.0 – 37.1
Georgia	Franklin	Non-metro	8	Carnesville	2,317	16.2	9.3 – 25.1
Alabama	Jefferson	Metro	1	Birmingham	37,181	15.9	11.9 – 20.9
65 – 74 Lowest Mortality							
New York	Suffolk	Metro	1	Brookhaven	120,833	1.0	0.7 – 1.4
District of Columbia	District of Columbia	Metro	1	Washington	17,268	1.0	0.5 – 1.7
Florida	Palm Beach	Metro	1	Jupiter, Palm Beach Gardens, Wellington, and West Palm Beach	126,247	0.9	0.6 – 1.3
New York	Westchester	Metro	1	New Rochelle, Scarsdale, Tarrytown, White Plains, and Yonkers	63,134	0.8	0.5 – 1.2
New York	Bronx	Metro	1	New York - Bronx	17,183	0.7	0.4 – 1.2
New York	Richmond	Metro	1	New York - Staten Island	34,877	0.7	0.4 – 1.0
New York	New York	Metro	1	New York - Manhattan	87,907	0.6	0.4 – 0.9
New York	Nassau	Metro	1	Hempstead	102,193	0.6	0.4 – 0.9
New York	Queens	Metro	1	New York - Queens	71,033	0.5	0.3 – 0.8
New York	Kings	Metro	1	New York - Brooklyn	82,949	0.4	0.2 – 0.7

The highest protein-energy malnutrition mortality thresholds were chosen based on the 99th percentile of mortality for each age group, and lowest based on the 1st percentile of mortality for each age group. For the age group ≥ 75 years old, the highest (99th) threshold was ≥ 154.0 per 100,000 and the lowest (1st) threshold was ≤ 12.3. For the age group 65-74 years old, the highest (99th) threshold was ≥ 15.8 per 100,000 deaths and the lowest (1st) was ≤ 1.1.

Rural-Urban continuous code: 1 - counties in metro areas of 1 million population or more; 2 – counties in metro areas of 250,000 to 1 million population; 3 – counties in metro areas fewer than 250,000 population; 4 - urban population of 20,000 or more, adjacent to a metro area; 5 - urban population of 20,000 or more, not adjacent to a metro area; 6 - urban population of 5,000 to 20,000 adjacent to a metro area; 7 - urban population of 5,000 to 20,000 not adjacent to a metro area; 8 - urban population of fewer than 5,000, adjacent to a metro area. Population: Estimated population for the age bracket, race/ethnicity, and county.

SUPPLEMENTAL TABLE S6. Counties with the Highest and Lowest Protein-Energy Malnutrition Mortality for the American Indian/Alaskan Native Population by Age Group in 2019

State	County	Rural Urban Classification	Rural-Urban Continuous Code	Main cities	Population (n)	Mortality (deaths per 100,000)	Uncertainty Interval
≥ 75 Highest Mortality							
Montana	Cascade	Metro	3	Great Falls	146	254.7	102.9 – 521.3
Arizona	Gila	Non-metro	4	Globe and Payson	277	170.6	84.7 – 303.2
Arizona	Coconino	Metro	3	Flagstaff	1,395	165.7	102.6 – 245.5
North Carolina	Scotland	Non-metro	6	Laurinburg	138	161.6	66.1 – 320.5
≥ 75 Lowest Mortality							
Maryland	Montgomery	Metro	1	Bethesda, Gaithersburg, Rockville and Silver Spring	329	11.0	3.6 – 26.1
New York	New York	Metro	1	New York - Manhattan	222	10.6	2.4 – 32.4
Florida	Miami-Dade	Metro	1	Doral, Hialeah, Homestead, Miami	156	10.0	1.4 – 34.6
Florida	Broward	Metro	1	Fort Lauderdale	194	9.6	2.1 – 26.0
New York	Westchester	Metro	1	New Rochelle, Scarsdale, Tarrytown, White Plains, and Yonkers	116	9.1	1.9 – 27.0
New York	Kings	Metro	1	New York - Brooklyn	340	8.8	1.9 – 26.1
New York	Nassau	Metro	1	Hempstead	118	8.5	1.7 – 27.2
Florida	Palm Beach	Metro	1	Jupiter, Palm Beach Gardens, Wellington, and West Palm Beach	140	6.8	1.4 – 19.9
New York	Queens	Metro	1	New York - Queens	524	5.5	1.1 – 15.1
65-74 Highest Mortality							
Montana	Cascade	Metro	3	Great Falls	261	34.9	13.1 – 72.0
North Carolina	Scotland	Non-metro	6	Laurinburg	345	24.2	9.6 – 46.9
South Dakota	Minnehaha	Metro	2	Sioux Falls	81	22.8	8.5 – 48.4
Arizona	Gila	Non-metro	4	Globe and Payson	499	20.1	8.5 – 36.7
Colorado	Pueblo	Metro	3	Pueblo and Colorado Springs	146	19.2	8.3 – 37.0
Louisiana	Lafourche Parish	Metro	3	Thibodaux	173	17.2	6.6 – 35.8
North Carolina	Cumberland	Metro	2	Fayetteville	350	16.8	7.3 – 32.8

Louisiana	Saint Tammany Parish	Metro	2	Covington, Slidell, and New Orleans	124	16.8	6.8 – 34.8
Alaska	Anchorage Municipality	Metro	2	Anchorage	1,625	16.6	6.6 – 32.0
65-74 Lowest Mortality							
New York	Kings	Metro	1	New York - Brooklyn	519	1.0	0.2 – 3.0
New York	Nassau	Metro	1	Hempstead	154	0.9	0.2 – 2.7
Florida	Palm Beach	Metro	1	Jupiter, Palm Beach Gardens, Wellington, and West Palm Beach	275	0.8	0.2 – 2.3
New York	Queens	Metro	1	New York - Queens	747	0.6	0.1 – 1.8

The highest protein-energy malnutrition mortality thresholds were chosen based on the 99th percentile of mortality for each age group, and lowest based on the 1st percentile of mortality for each age group. For the age group ≥ 75 years old, the highest (99th) threshold was ≥ 154.0 per 100,000 and the lowest (1st) threshold was ≤ 12.3. For the age group 65-74 years old, the highest (99th) threshold was ≥ 15.8 per 100,000 deaths and the lowest (1st) was ≤ 1.1.

Rural-Urban continuous code: 1 - counties in metro areas of 1 million population or more; 2 – counties in metro areas of 250,000 to 1 million population; 3 – counties in metro areas with fewer than 250,000 population; 4 - urban population of 20,000 or more, adjacent to a metro area; 6 - urban population of 5,000 to 20,000 adjacent to a metro area; Population: Estimated population for the age bracket, race/ethnicity, and county.

SUPPLEMENTAL TABLE S7. Counties with the Highest and Lowest Protein-Energy Malnutrition Mortality for the Latino Population by Age Group in 2019

State	County	Rural Urban Classification	Rural-Urban Continuous Code	Main cities	Population (n)	Mortality (deaths per 100,000)	Uncertainty Interval
≥ 75 Highest Mortality							
New Mexico	Bernalillo	Metro	2	Albuquerque	15,400	173.1	138.5 – 211.3
New Mexico	Cibola and Valencia	Non-metro	6	Grants and Los Lunas	3,052	172.9	126.5 – 228.0
Utah	Carbon	Non-metro	7	Price	139	172.0	73.4 – 314.4
Colorado	Pueblo	Metro	3	Pueblo and Colorado Springs	3,545	165.3	110.3 – 226.4
Colorado	Otero	Non-metro	4	La Junta	458	161.8	83.9 – 277.4
New Mexico	Chaves	Non-metro	5	Roswell	1,476	156.7	95.7 – 228.1
≥ 75 Lowest Mortality							
Virginia	Loudoun	Metro	1	Leesburg	887	12.3	5.4 – 24.1
District of Columbia	District of Columbia	Metro	1	Washington	1,696	12.3	4.5 – 25.7
Maryland	Frederick	Metro	1	Frederick	433	11.6	4.3 – 25.6
Florida	Miami-Dade	Metro	1	Doral, Hialeah, Homestead, Miami	138,576	11.4	8.6 – 14.8
New York	Putnam	Metro	1	Carmel Hamlet	392	11.4	3.8 – 25.7
Florida	Saint Lucie	Metro	2	Fort Pierce and Port Saint Lucie	2,623	10.9	5.1 – 19.1
Massachusetts	Norfolk	Metro	1	Norfolk, Dedham, Norwood	849	10.8	4.4 – 22.4
New Jersey	Hudson	Metro	1	Jersey City, Hoboken	15,172	10.2	5.7 – 16.7
Maryland	Howard	Metro	1	Columbia	401	9.9	3.5 – 22.9
Rhode Island	Washington	Metro	1	South Kingstown	93	9.9	2.7 – 25.7
Florida	Broward	Metro	1	Fort Lauderdale	27,458	9.9	5.8 – 15.7
New York	Suffolk	Metro	1	Brookhaven	7,563	9.8	4.8 – 17.7
Rhode Island	Bristol	Metro	1	Bristol	87	9.8	2.5 – 26.7
New Jersey	Bergen	Metro	1	Hackensack	6,857	9.7	4.9 – 17.2
New York	Richmond	Metro	1	New York - Staten Island	2,612	8.3	3.7 – 17.1
Florida	Palm Beach	Metro	1	Jupiter, Palm Beach Gardens, Wellington,	14,583	7.9	4.1 – 13.4

				and West Palm Beach			
New York	Westchester	Metro	1	New Rochelle, Scarsdale, Tarrytown, White Plains, and Yonkers	7,770	7.0	3.5 – 12.3
Florida	Martin	Metro	2	Palm city, Stuart	604	6.3	2.7 – 11.9
New York	New York	Metro	1	New York - Manhattan	29,967	5.7	3.1 – 9.1
New York	Bronx	Metro	1	New York - Bronx	36,967	5.4	3.1 – 8.6
New York	Nassau	Metro	1	Hempstead	7,609	5.1	2.3 – 9.4
New York	Kings	Metro	1	New York - Brooklyn	22,221	4.8	2.3 – 8.7
New York	Queens	Metro	1	New York - Queens	28,435	4.2	2.3 – 7.0
65-74 Lowest Mortality							
Virginia	Loudoun	Metro	1	Leesburg	1,807	1.1	0.5 – 2.0
Rhode Island	Washington	Metro	1	Narragansett, Westerly	150	1.1	0.3 – 2.8
Massachusetts	Norfolk	Metro	1	Norfolk, Dedham, Norwood	1,246	1.0	0.4 – 2.2
Maryland	Montgomery	Metro	1	Bethesda, Gaithersburg, Rockville and Silver Spring	14,374	1.0	0.6 – 1.7
Florida	Miami-Dade	Metro	1	Doral, Hialeah, Homestead, Miami	158,678	1.0	0.6 – 1.6
New York	Rockland	Metro	1	Nyack, New City, Ramapo	2,580	1.0	0.4 – 2.0
California	Los Angeles	Metro	1	Los Angeles, Beverly Hills, Santa Monica, Long Beach	261,579	1.0	0.7 – 1.4
Maryland	Frederick	Metro	1	Frederick	817	1.0	0.4 – 2.0
New York	Putnam	Metro	1	Carmel Hamlet	665	0.9	0.3 – 2.1
New York	Suffolk	Metro	1	Brookhaven	12,677	0.9	0.5 – 1.7
Maryland	Howard	Metro	1	Columbia	687	0.9	0.4 – 1.9
District of Columbia	District of Columbia	Metro	1	Washington	3,073	0.9	0.3 – 2.1
New Jersey	Bergen	Metro	1	Hackensack	10,576	0.9	0.5 – 1.5
New Jersey	Hudson	Metro	1	Jersey City, Hoboken	20,145	0.9	0.5 – 1.4

New York	Richmond	Metro	1	New York - Staten Island	4,386	0.8	0.4 – 1.6
Florida	Martin	Metro	2	Palm city, Stuart	989	0.8	0.3 – 1.6
Florida	Broward	Metro	1	Fort Lauderdale	40,150	0.8	0.4 – 1.3
Florida	Palm Beach	Metro	1	Jupiter, Palm Beach Gardens, Wellington, and West Palm Beach	20,600	0.7	0.3 – 1.2
New York	Westchester	Metro	1	New Rochelle, Scarsdale, Tarrytown, White Plains, and Yonkers	12,159	0.6	0.3 – 1.1
New York	Bronx	Metro	1	New York - Bronx	51,277	0.5	0.3 – 0.9
New York	New York	Metro	1	New York - Manhattan	35,203	0.5	0.3 – 0.9
New York	Nassau	Metro	1	Hempstead	12,133	0.5	0.2 – 0.8
New York	Kings	Metro	1	New York - Brooklyn	32,880	0.5	0.2 – 0.9
New York	Queens	Metro	1	New York - Queens	43,242	0.4	0.2 – 0.7

The highest protein-energy malnutrition mortality thresholds were chosen based on the 99th percentile of mortality for each age group, and lowest based on the 1st percentile of mortality for each age group. For the age group ≥ 75 years old, the highest (99th) threshold was ≥ 154.0 per 100,000 and the lowest (1st) threshold was ≤ 12.3. For the age group 65-74 years old, the highest (99th) threshold was ≥ 15.8 per 100,000 deaths and the lowest (1st) was ≤ 1.1.

Rural-Urban continuous code: 1 - counties in metro areas of 1 million population or more; 2 – counties in metro areas of 250,000 to 1 million population; 3 – counties in metro areas fewer than 250,000 population; 4 - urban population of 20,000 or more, adjacent to a metro area; 5 - urban population of 20,000 or more, not adjacent to a metro area; 6 - urban population of 5,000 to 20,000 adjacent to a metro area; 7 - urban population of 5,000 to 20,000 not adjacent to a metro area. Population: Estimated population for the age bracket, race/ethnicity, and county.

SUPPLEMENTAL TABLE S8. Counties with the Lowest Protein-Energy Malnutrition Mortality for the Asian Population by Age Group in 2019

State	County	Rural Urban Classification	Rural-Urban Continuous Code	Main cities	Population (n)	Mortality (deaths per 100,000)	Uncertainty Interval
≥ 75 Lowest Mortality							
New York	Putnam	Metro	1	Carmel Hamlet	124	11.9	3.5 – 31.3
California	Los Angeles	Metro	1	Los Angeles, Beverly Hills, Santa Monica, Long Beach	121,102	11.6	8.6 – 15.3
California	Santa Clara	Metro	1	San Jose	40,125	11.5	7.5 – 16.4
Hawaii	Kauai	Nonmetro	5	Kapa'a	3,550	11.0	4.3 – 21.7
Hawaii	Hawaii	Nonmetro	5	Kailua-Kona, Hilo	8,307	10.5	4.4 – 20.2
Florida	Palm Beach	Metro	1	Jupiter, Palm Beach Gardens, Wellington, and West Palm Beach	2,287	10.5	4.3 – 21.0
New York	Rockland	Metro	1	Nyack, New City, Ramapo	1,586	10.2	3.9 – 21.6
Florida	Saint Lucie	Metro	2	Fort Pierce and Port Saint Lucie	422	9.9	3.4 – 22.4
New Jersey	Bergen	Metro	1	Hackensack	9,192	8.7	4.6 – 15.1
New Jersey	Hudson	Metro	1	Jersey City, Hoboken	3,893	8.6	4.6 – 15.1
Hawaii	Kalawao and Maui	Nonmetro	3	Kalaupapa, Kahului	6,734	7.4	2.8 – 14.7
New York	Westchester	Metro	1	New Rochelle, Scarsdale, Tarrytown, White Plains, and Yonkers	3,588	6.8	3.2 – 12.3
Florida	Martin	Metro	2	Palm city, Stuart	168	6.5	2.2 – 14.4
New York	Bronx	Metro	1	New York - Bronx	2,918	6.2	2.9 – 12.3
New York	Richmond	Metro	1	New York - Staten Island	2,565	6.1	2.6 – 12.3
New York	Nassau	Metro	1	Hempstead	6,941	5.9	2.7 – 11.3
New York	New York	Metro	1	New York - Manhattan	14,179	4.8	2.4 – 8.3
New York	Kings	Metro	1	New York - Brooklyn	15,574	4.5	2.2 – 8.3
New York	Queens	Metro	1	New York - Queens	35,253	4.4	2.5 – 7.2
65-74 Lowest Mortality							

Rhode Island	Washington	Metro	1	Narragansett, Westerly	182	1.0	0.3 – 2.9
District of Columbia	District of Columbia	Metro	1	Washington	1,368	1.0	0.3 – 2.2
Maryland	Howard	Metro	1	Columbia	3,998	1.0	0.4 – 2.0
New Jersey	Essex	Metro	1	Newark	3,879	1.0	0.5 – 2.0
New Jersey	Middlesex	Metro	1	New Brunswick	14,267	1.0	0.5 – 1.9
Maryland	Frederick	Metro	1	Frederick	728	1.0	0.4 – 2.1
New Jersey	Morris	Metro	1	Morristown	4,237	1.0	0.5 – 1.9
Hawaii	Kauai	Nonmetro	5	Kapa'a	4,338	1.0	0.3 – 2.5
New York	Dutchess	Metro	2	Poughkeepsie	1,049	1.0	0.4 – 2.0
New Jersey	Hunterdon	Metro	1	Flemington, Clinton	522	1.0	0.3 – 2.3
Hawaii	Hawaii	Nonmetro	5	Kailua-Kona, Hilo	11,822	1.0	0.3 – 2.3
New Jersey	Somerset	Metro	1	Somerville, Bridgewater	4,152	0.9	0.4 – 1.9
New York	Putnam	Metro	1	Carmel	146	0.9	0.3 – 2.0
California	Los Angeles	Metro	1	Los Angeles, Beverly Hills, Santa Monica, Long Beach	163,139	0.8	0.5 – 1.3
California	Santa Clara	Metro	1	San Jose	55,936	0.8	0.5 – 1.3
Florida	Palm Beach	Metro	1	Jupiter, Palm Beach Gardens, Wellington, and West Palm Beach	4,190	0.8	0.3 – 1.7
New York	Suffolk	Metro	1	Brookhaven	4,668	0.8	0.4 – 1.5
New York	Rockland	Metro	1	Nyack, New City, Ramapo	2,320	0.8	0.3 – 1.6
Florida	Martin	Metro	2	Palm city, Stuart	203	0.8	0.2 – 1.7
New Jersey	Bergen	Metro	1	Hackensack	13,315	0.7	0.4 – 1.2
Hawaii	Kalawao and Maui	Nonmetro	3	Kalaupapa, Kahului	9,002	0.7	0.2 – 1.6
New Jersey	Hudson	Metro	1	Jersey City, Hoboken	6,648	0.7	0.3 – 1.3
New York	Bronx	Metro	1	New York - Bronx	4,911	0.5	0.2 – 1.1
New York	Richmond	Metro	1	New York - Staten Island	4,991	0.5	0.2 – 1.1
New York	Westchester	Metro	1	New Rochelle, Scarsdale, Tarrytown, White Plains, and Yonkers	5,338	0.5	0.2 – 0.9
New York	Nassau	Metro	1	Hempstead	12,258	0.4	0.2 – 0.8

New York	New York	Metro	1	New York - Manhattan	16,360	0.4	0.2 – 0.8
New York	Kings	Metro	1	New York - Brooklyn	26,010	0.4	0.2 – 0.8
New York	Queens	Metro	1	New York - Queens	58,513	0.4	0.2 – 0.6

The highest protein-energy malnutrition mortality thresholds were chosen based on the 99th percentile of mortality for each age group, and lowest based on the 1st percentile of mortality for each age group. For the age group ≥ 75 years old, the highest (99th) threshold was ≥ 154.0 per 100,000 and the lowest (1st) threshold was ≤ 12.3. For the age group 65-74 years old, the highest (99th) threshold was ≥ 15.8 per 100,000 deaths and the lowest (1st) was ≤ 1.1.

Rural-Urban continuous code: 1 - counties in metro areas of 1 million population or more; 2 – counties in metro areas of 250,000 to 1 million population; 3 – counties in metro areas fewer than 250,000 population; 5 - urban population of 20,000 or more, not adjacent to a metro area.
